# Midline vs. lateral flank approach for spaying nutrias (*Myocastor coypus*)

**DOI:** 10.3389/fvets.2025.1529359

**Published:** 2025-01-29

**Authors:** Francesco Di Ianni, Igor Pelizzone, Martina Gavezzoli, Martina Fumeo, Alessandro Vetere

**Affiliations:** Department of Veterinary Science, University of Parma, Parma, Italy

**Keywords:** *Myocastor coypus*, nutria, spaying, wildlife population control, exotic animal medicine

## Abstract

The nutria (*Myocastor coypus*), an invasive semi-aquatic rodent in Europe introduced for the fur and meat industry at the beginning of the 20^th^ century, has rapidly become a significant ecological and economic concern. In Italy, the damage caused by nutrias to crops, wetlands, and drainage systems has prompted the development of containment plans. However, these efforts, while effective in the short term, are challenged by rapid recolonization and local resistance. One emerging approach for controlling nutria populations is sterilization. This study compared two surgical techniques for ovariectomy in 60 free-ranging nutrias: 30 animals underwent the flank approach (FA), and 30 underwent the ventral midline approach (VMA). The animals were randomly assigned to one of the two groups and monitored for anesthesia duration, surgery time, recovery, and return to feeding. Results indicated that the FA group exhibited significantly shorter surgical and recovery times than the VMA group, with a positive correlation between body weight and surgery duration only in the VMA group. Additionally, a higher percentage of animals in the FA group resumed feeding within 12 h, suggesting better post-operative recovery. The FA technique thus demonstrated advantages over the VMA, reducing the risk of intraoperative complications and shortening recovery times. These findings suggest that the FA technique may be more suitable for reproductive control of nutria as part of invasive wildlife management strategies.

## Introduction

The nutria (*Myocastor coypus*), a semiaquatic rodent native to South America, has become a major invasive species across Europe and North America (1). Originally brought over for meat and fur production, many nutrias were released into the wild after the fur industry declined (1). Their high reproductive rate and adaptability have enabled them to establish populations in various environments, particularly in Europe and Italy, where they were first introduced in 1928 (2). The spread of nutria has resulted in serious ecological and economic impacts since these rodents cause significant damage to crops, wetlands, and drainage systems (3–6). Their burrowing activities destabilize rice fields and riverbanks, increasing erosion and flood risks (6). Nutria also compete with native species, including birds, for resources, thereby disrupting local ecosystems (5, 6). In response to these issues, many countries have classified nutria as pests, leading to the development of various containment strategies (7–10). The European Union has designated nutria as an “invasive alien species of Union concern” under Commission Implementing Regulation (EU) no. 2016/1141. This designation, supported by regulation (EU) no. 1143/2014 and Italy's D.L. 230/17 concerning invasive species management, has prompted the development of national plans to monitor, contain, and eliminate nutria populations (11). While containment plans have had some success, they have not provided a lasting solution, as cleared areas are quickly recolonized (5, 6, 11). Furthermore, these efforts are often compromised by public resistance and logistical difficulties, especially in urban areas. Consequently, numerous research projects have focused on studying their distribution, behavior, and environmental impacts. In Italy, although regional containment plans aim to reduce economic damage and protect biodiversity, the long-term success of such plans may be stifled by rapid recolonization (11–15). Public opposition to large-scale culling and the complexity of implementing such measures in urban settings further complicate control efforts (1, 13). An alternative approach gaining popularity is population control through sterilization, which is often more publicly acceptable. For this method to be effective, it is crucial to conduct a comprehensive evaluation of the necessary measures, including cost analysis, compliance with current legislation, sustainability, effectiveness, and efficiency (1, 3, 4, 6, 11, 13, 14, 16, 17). In a recent study, Bonaffini et al. (11) spayed 77 nutrias under general anesthesia using two different methods. Thirty-two patients underwent laparoscopic salpingectomies and vasectomies, and the others underwent traditional surgeries, keeping their gonads intact. In that study, traditional surgery was faster than laparoscopic surgery (*p* = 0.004), but both methods showed low complication rates. Postoperative monitoring revealed no signs of infection, wound dehiscence, or social behavior changes, with all animals resuming normal activities quickly after release. However, two mortalities due to peritonitis occurred in the traditional surgery group. The animals were released without complications or behavioral changes, and the population decreased in the following months (11).

In this study, we compared the flank and midline spaying techniques in 62 free-ranging nutrias (*Myocastor coypus*).

## Methods

### Animals

The study was approved by the University of Parma (PROT. N. 13/CESA/2024). The exclusion criteria included any history or visible signs of systemic illness (e.g., signs of infection, external wounds, inflammation, or trauma). Sixty-two non-pregnant female free-ranging nutrias were presented for spaying as part of an invasive population management project. Each animal was captured the night before surgery by trained volunteers using apples as bait inside a trap cage. The procedures took place from September 2023 to August 2024. After capture, they were transported early in the morning to the hospital facility and kept in the dark for 4 h to acclimatize. All the animals were adults with no overt health issues based on physical inspection. When an animal was presented, it was randomly assigned (www.randomizer.org, accessed on 25/08/2024) to either the ventral midline approach (VMA) group or the flank approach (FA) group. Thirty animals were assigned to the VMA group, and 30 were assigned to the FA group. Two animals were excluded from the study upon arrival at the facility because of traumatic fracture of the incisor teeth.

### Perioperative management

Food (mixed vegetables) or water were not withheld prior to anesthesia induction. All the animals were initially weighed using trap cages with standard weight and were intramuscularly administered 50 μg/kg dexmedetomidine (Dexdomitor 0.5 mg/ml, Vétoquinol Italia S.r.l., Bertinoro, Italy), 5 mg/kg ketamine (Lobotor 10 mg/100 ml, Acme S.r.l., Cavriago, Italy), and 0.3 mg/kg methadone (Semfortan 10 mg/ml, Dechra Veterinary Products S.r.l., Torino, Italy). After sedation, the animals were preoxygenated via a facemask, and an ultrasound examination of the genital system was performed to assess the clinical status of the reproductive tract and to locate the ovaries precisely.

**Figure 1:**
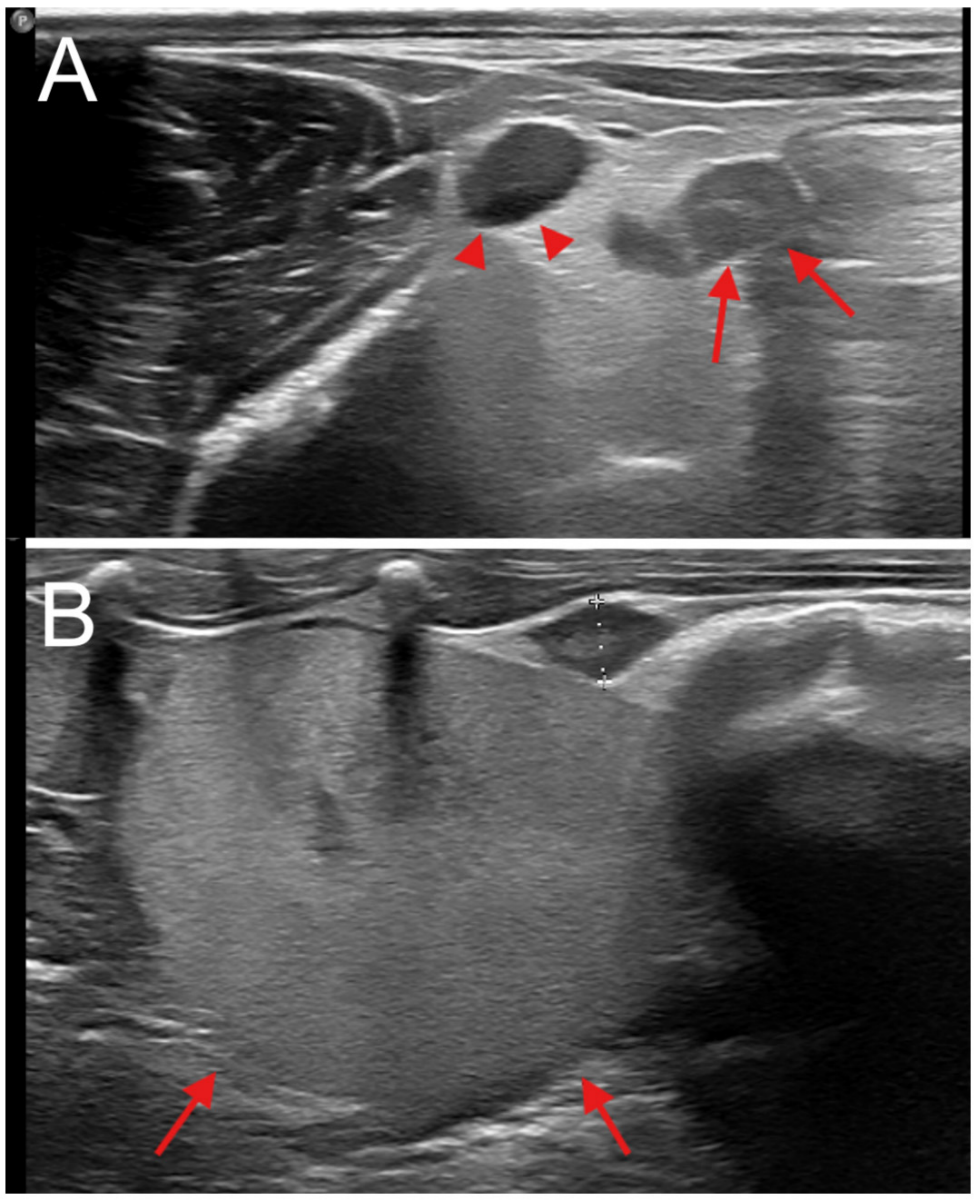
Abdominal ultrasound. **(A)** Presumed post-partum, transverse view, dorsal approach; enlarged ovary, indicated by arrows, lateral to a large vessel, supposedly of uterine origin, indicated by arrowheads. **(B)** Sagittal view, dorsal approach; normal ovary (oval hypoechoic structure between calipers), caudal to the kidney (indicated by arrows).

All abdominal ultrasonographic examinations (Panther, Esaote, Genoa - Italy) were performed using a linear high-frequency transducer (L15-7 MHz). In the group of animals where the flank approach was carried out, the ultrasound was performed in sternal recumbency, shaving two square areas of approximately 6 cm^2^ starting from the last rib in a caudal direction and 2 cm from the spine in a ventral direction (Figures 1A, B). In the group of animals where the ventral approach was performed, the ultrasound was carried out in dorsal recumbency with standard shaving for an abdominal ultrasound. The abdominal ultrasound also revealed the presence of pregnant animals, which were excluded from the study. Following the ultrasound examination, an IV catheter (Delta Med S.p.a, via Guido Rossa 20, Viadana, (MN) 46019, Italy) was placed in the right cephalic vein, and warmed crystalloid fluids (Lactated Ringer's Solution) were administered at a rate of 10 ml/kg/h. Animals were induced via mask with 3% to 5% isoflurane (IsoFlo 250 ml, Zoetis Italia S.r.l., Roma, Italy) with 100% oxygen at a flow rate of 1 to 2 L/min with pediatric Bain circuit, and maintenance was performed via mask with 2% isoflurane and 100% oxygen at a flow rate of 0.5–1 L/min. The surgical sites were aseptically prepared with 2.0% chlorhexidine digluconate (Clorexinal 2%, Nuova Farmec, Via Walther Fleming 7, Settimo, VR, Italy). The animals were maintained on heating pads throughout the surgical procedure and their rectal temperature was monitored. Heart rate via ECG, non-invasive blood pressure, peripheral oxygen saturation, and respiratory rate were continuously monitored. The respiratory rate was visually monitored. Postoperatively, all the animals received 0.5 mg/kg meloxicam subcutaneously (Meloxoral 5 mg/ml, A.T.I. Azienda Terapeutica Veterinaria S.r.l., Ozzano dell'Emilia, Italy) and 30 μg/kg buprenorphine intramuscularly (Bupaq Multidose 0.3 mg/ml, LIVISTO Company, Modena, Italy) to provide appropriate analgesia.

All nutrias were individually identified with a subcutaneous electronic transponder (TheraPet Transponder NANO CHIP 1.4 × 8.5 mm, Bioforlife Italia srl,). At the end of the procedure, atipamezole (0.5 mg/kg; Antisedan 5 mg/mL, Vétoquinol Italia S.r.l.) was administered intramuscularly as a reversal agent. The recovery time was measured from the time of reversal agent administration, which coincided with the discontinuation of isoflurane, until the resumption of the standing position.

**Figure 2:**
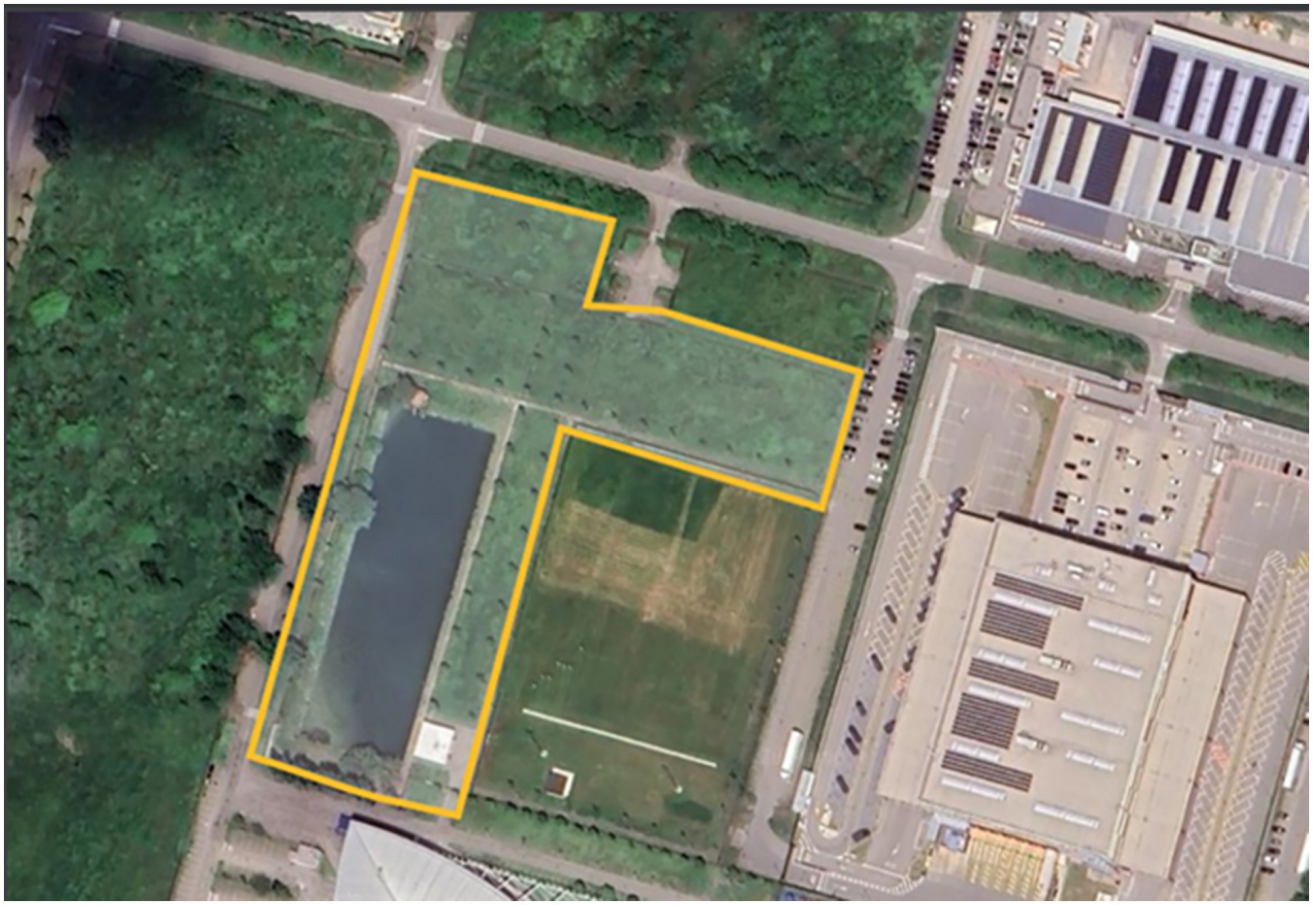
Sanctuary's floor plan. The total area of the sanctuary is 18,000 m^2^, with the lake occupying a surface of 4,000 m^2^.

After surgery, the animals were housed individually for approximately 12 h in metal cages measuring approximately 60 × 60 × 50 cm, with access to water and food consisting of chopped fresh vegetables (radish, carrots, and dandelion). After this period, the animals were released into a sanctuary (Figure 2) where they could interact with other sterilized rodents, providing a more natural and enriching habitat while still being monitored for any postoperative complications or signs of discomfort.

### Surgical approach

#### Flank approach surgical technique

**Figure 3:**
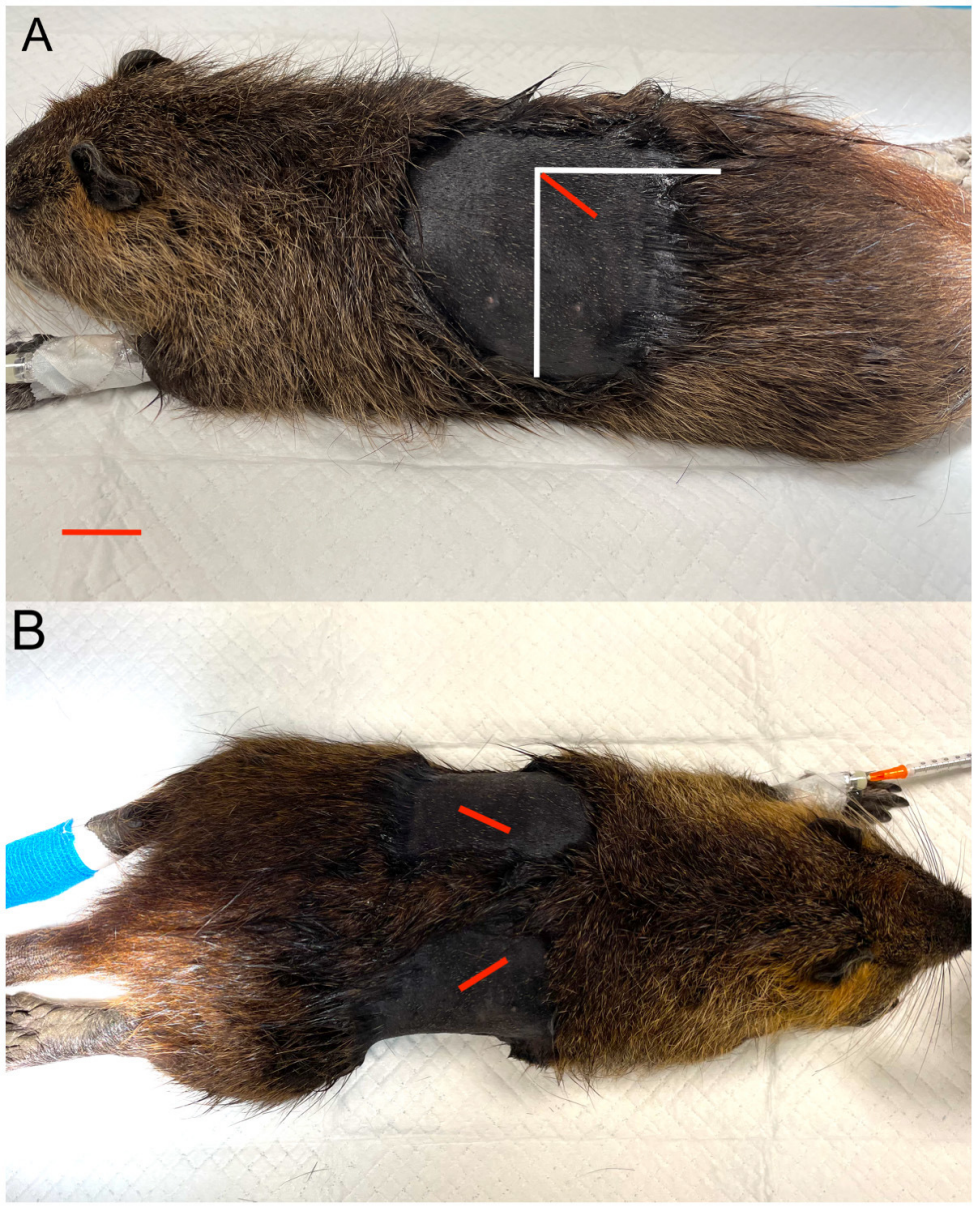
Ovarian localization and incision sites. **(A)** Lateral recumbency. **(B)** Dorsoventral recumbency. The red line indicates the incision site; the vertical and horizontal white lines represent, respectively, the caudal edge of the last rib and the lateral edge of the lumbar vertebral transverse processes.

**Figure 4:**
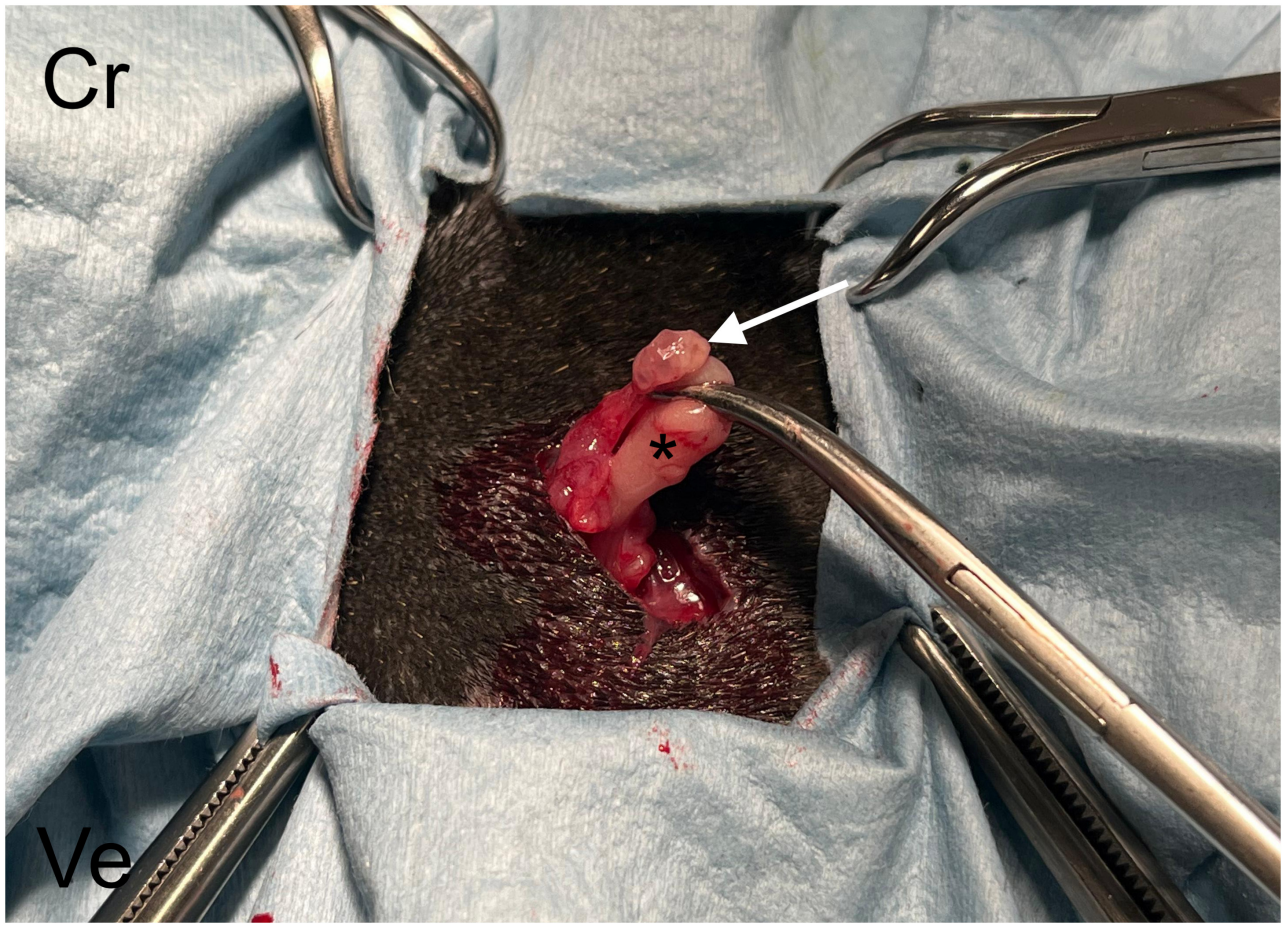
Surgical procedure. The left ovary (white arrow) and the ipsilateral proximal oviduct (asterisk) are exteriorized through the surgical breach. Cr, cranial; Ve, ventral.

Patients were placed in ventral recumbency, and the bilateral flank regions were clipped and aseptically prepared. In accordance with what was shown during echography, a 1–2 cm skin incision was made caudally to the border of the last rib, just ventrally to the vertebral transverse process. The right ovary was localized more cranially with respect to the left ovary, so the right incision was made cranio-caudally and dorso-ventrally from the last rib to the transverse process of the II lumbar vertebra (Figures 3A, B) (18). The left incision was made 1 cm caudal the right one. Blunt dissection of the external and internal abdominal oblique muscles was performed with Metzenbaum scissors, and the peritoneum was incised; the uppermost ovary or uterine horn was then located within the exposed area (Figure 4). This fat was shifted caudally, then the uterine horn was manipulated to expose the ovary. Hemostatic forceps were applied caudal to the ovary and oviduct to incorporate the ovarian vessels. A 3/0 glyconate sutures (Monosyn^®^ Braun Avitum Italy S.p.A. Mirandola, Italy) were used to construct a single Miller's knot proximal to the hemostatic clamp and the ovary and oviduct were completely removed. The clamp was subsequently removed, and the remaining uterine tissue was retracted back into the peritoneal cavity. The body wall was closed with 2/0 glyconate adsorbable continuous pattern sutures (Monosyn^®^ Braun Avitum Italy S.p.A. Mirandola, Italy), and the skin was closed with intradermal pattern using 3/0 glyconate sutures (Monosyn^®^ Braun Avitum Italy S.p.A. Mirandola, Italy).

#### Ventral midline approach

Patients were placed in dorsal recumbency, and the ventral abdomen was aseptically prepared. A 5 to 8 cm skin incision was made along the ventral midline between the umbilicus and the pubis. The subcutaneous tissue and the Linea Alba were dissected to gain access to the abdominal cavity. The abdominal viscera were gently retracted cranially to locate the uterine horns. The uterine horns were exteriorized to expose the ovary and the short suspensory ligament attachment. Additional dissection of the ovarian vessels and periovarian fat was occasionally necessary. At this point, the ovarian suspensory ligament was isolated and then ligated using 3/0 absorbable sutures (Monosyn^®^, Braun Avitum Italy S.p.A., Mirandola, Italy) before the mesovarium and vessels were transected distally. Another ligature was placed distally on the ovary before its complete removal. This process was repeated for the contralateral ovary. The Linea Alba was closed with a 3-0 polyglactin 910 absorbable suture (Vicryl^®^ 2/0, Johnson & Johnson, Italy) in a simple suture pattern. The subcutis and the skin were closed with an intradermal pattern using 3/0 glyconate suture (Monosyn^®^ Braun Avitum Italy S.p.A. Mirandola, Italy).

### Statistical analysis

The anesthesia time, surgery time, recovery time, and total procedure time were recorded for both groups. The anesthesia time was defined as the time from the initial administration of isoflurane to the time the isoflurane vaporizer was turned to 0%. The surgery time was defined as the time from the first incision to the time the final skin suture was placed. The recovery time was defined as the time when the isoflurane vaporizer was turned off (0%), which coincided with the administration of the reversal agent to when the animal fully recovered the standing position. The procedure time was defined as the time from the initial activation time of isoflurane to the time of the first spontaneous movement of the animal.

Statistical analyses were conducted in R v 4.4.1 to evaluate the effects of weight (classified into three classes: low, medium, and high based on percentiles) and type of surgery (FA and VMA), as well as their interaction, on several dependent variables: anesthesia time, surgery time, recovery time, and total time. Linear models were fitted for each dependent variable using weight class, type, and their interaction as fixed effects. ANOVA was performed to assess the significance of the fixed effects, with F-values and *p*-values reported. Factors were evaluated as significant if *p*-value was ≤ 0.05. Least-squares means (LSM) were computed for each combination of weight class and type to estimate marginal means and associated standard errors.

Diagnostic plots, including residuals vs. fitted values, normal Q-Q plots, scale-location plots, and residuals vs. leverage plots, were generated to assess model assumptions such as linearity, homoscedasticity, and normality of residuals.

To visually represent the results, bar plots of the LSM were created, with bars indicating the standard error and the mean represented as dots.

## Results

Animals' weights ranged from 2.8 kg (6.2 lbs) to 5.7 kg (12.6 lbs) [mean 4.3 kg (9.5 lbs)].

In the VMA group, 2/30 (6.7%) patients suffered moderate hemorrhage secondary to the rupture of the right ovarian ligament during isolation and vascular ligation. For one patient, the time to identify the ovarian stump and stop the hemorrhage was approximately 4 min. In the second patient, more than 5 min were needed to recover and ligate the torn stump, as the intestinal mass made its location and manipulation difficult. No deaths were recorded during the surgical procedure in either group.

The animals were visually monitored at 1 week, 2 weeks, and 1 month post-surgery. No complications were observed.

**Figure 5:**
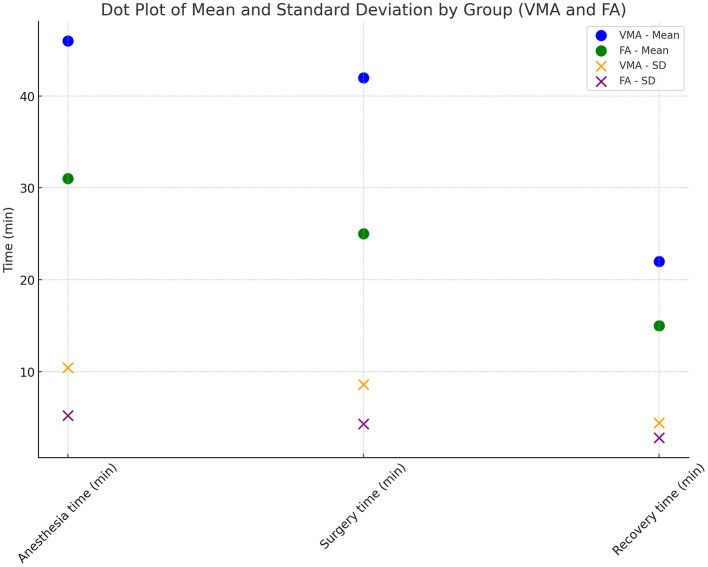
Mean times. Dot plot of mean times and standard deviation by groups (VMA and FA).

**Figure 6:**
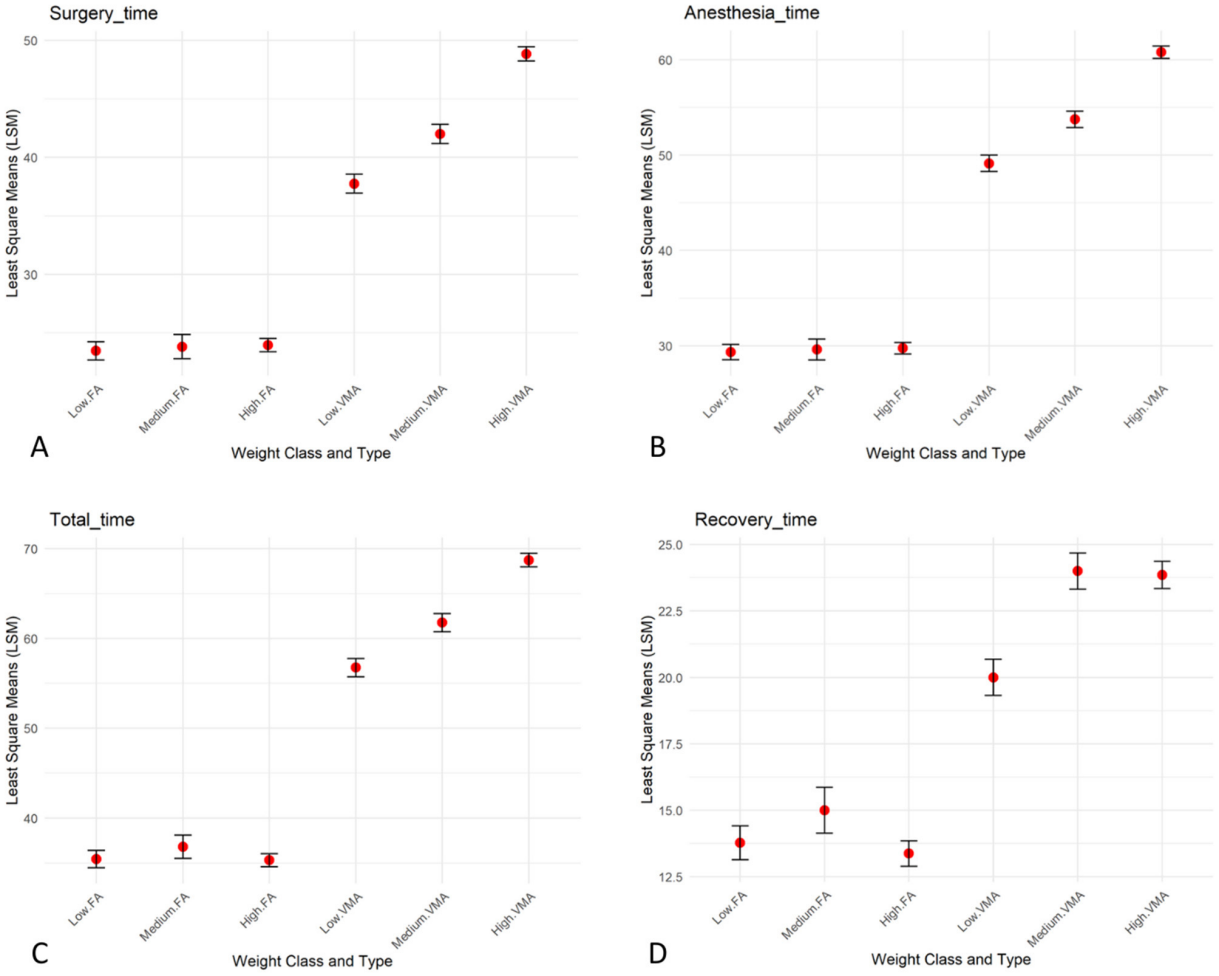
Group comparisons. Bar plots of the LSM, with bars indicating the standard error and the mean represented as dots showing the interaction between weight ang surgery approach (FA and VMA) for surgery time **(A)**, anesthesia time **(B)**, recovery time **(C)**, and total time **(D)**.

The statistical analysis of the data revealed that surgery time differed significantly based on the type of surgery and the interaction between Type and Weight, showing a longer surgery time with the VMA approach compared to the FA approach (Figures 5, 6A). Anesthesia time was significantly different with one group (VMA) requiring significantly more time under anesthesia (Figures 5, 6B). The mean anesthesia time in the VMA group was 55 min in the FA group 29 min. The recovery time also significantly differed; the animals in the FA group recovered faster than for those in the VMA group (Figures 5, 6C). Finally, the total procedure time was significantly different between the groups (Figures 5, 6D).

A total of 21/30 (70%) patients in the FA group and 5/30 (16.7%) in the VMA group ate within 12 h postoperatively.

## Discussion

Surgical techniques in rodents are being adapted to conventional species protocols and are continuously evolving. Two surgical approaches have been described for nutria (11). Traditional laparotomy is a quick procedure that is effective for salpingectomy; however, accessing the ovaries has been challenging because of their very dorsal location near the kidneys (11). The endovideo laparoscopic approach has been proven effective and free of side effects, but it is more suitable for vasectomy and salpingectomy as it preserves the intraspecific relationships within family groups (11). Moreover, the anatomical conformation of the suborder Hystricomorpha causes surgical wounds to remain in close contact with the ground, and the laparotomy incision is often positioned too close to the ground, which may lead to dehiscence (19). In our case, ovariectomy was necessary to prevent territorial fights, as the animals were destined for forced cohabitation within the recovery area in the sanctuary. The lateral flank approach serves as an alternative to the traditional ventral midline method for ovariohysterectomy in dogs and cats (20, 21). It is particularly indicated in cases of excessive mammary development or when postoperative examination may be restricted (20, 22). The benefits of this technique include a reduced risk of evisceration if the wound dehisces and the ability to visually monitor the incision from a distance post-surgery and increased procedural efficiency (20, 22). Given the unique anatomy of nutrias, their intestinal structure and digestive physiology have been previously studied. Research indicates that, morphologically, the large intestine of nutrias is similar to that of other caviomorphs, particularly guinea pigs (22–25);it is voluminous and adapted to the strictly herbivorous diet of this species (25). For this reason, performing elective ovariectomy in these species can be challenging. The midline abdominal approach requires manipulation of the intestines, potentially causing postoperative issues such as pain, peristaltic problems, and inappetence, and intensive postoperative care with additional nutrition, fluids, and pain management would be necessary (26–28). Moreover, the presence of long uterine horns and a short ovarian suspensory ligament complicates access to and ligation of the ovarian vessels (29, 30). In the flank approach presented here, contact with gastrointestinal organs was minimized, and the nutrias recovered rapidly and without complications. In contrast, two patients sterilized using the midline approach exhibited intraoperative issues, such as moderate bleeding due to traumatic rupture of the ovarian pedicle.

The anesthesia time is a crucial factor in veterinary procedures, as prolonged anesthesia can increase the risk of complications, even in hystricomorph rodents, such as nutrias (31), and in guinea pigs (32, 33). Prolonged anesthesia can depress the central nervous system, reduce cardiovascular function, and impair thermoregulation (34, 35). In this study anesthesia time was longer in the VMA group; this finding could be associated with a higher risk of anesthesia-related complications, especially in wild subjects whose age and previous medical history are unknown, and in which a thorough pre-anesthetic evaluation is not feasible. The surgery time also varied significantly between the two groups. The mean surgery time for the VMA was 44 min, which was notably longer than the 23 min observed in the FA group. This difference was statistically significant, meaning the chance of this result occurring randomly is very low. The longer surgery times in the VMA group reflect a more complex surgical procedure in the VMA group than in the FA group. The recovery time significantly differed between the two groups when the same anesthetic protocol was used. The average recovery time of the FA group was 13 min, whereas that of the VMA group was considerably longer, at 22.87.

In this study, 21 out of 30 animals (70%) in the FA group and only 5 out of 30 animals (16.7%) in the VMA group resumed eating within 12 h postoperatively. This significant difference suggests that the animals in the FA group experienced less postoperative discomfort or pain. The resumption of eating is widely recognized in literature as a key indicator of post-surgical recovery and pain management. A decrease in appetite or water consumption is often considered a sign of pain or stress, and, in several studies, the return to normal feeding is used as a primary indicator of reduced discomfort (36–38). Therefore, the greater percentage of animals in the FA group that resumed eating within 12 h may indicate more effective pain control and better overall postoperative recovery than those in the VMA group.

Gonadectomy may be a viable option and has been shown to negatively impact social behavior in various species of rodents (39, 40). However, studies linking gonadectomy to changes in social behavior in free-ranging nutria colonies are lacking. Given that the sterilized animals were placed in a confined space for the purpose of environmental eradication, the interaction between the various subjects introduced into the sanctuary falls outside the scope of this study.

The correlation between body weight and surgery duration in animals is an important consideration in veterinary surgery and is influenced by various physiological and technical factors. Previous studies in veterinary medicine have shown that greater body weight can present a range of challenges during surgical procedures, including increasing the surgical time needed for incisions and tissue manipulation, as well as ensuring adequate exposure of internal organs (41, 42). In rodents, such as nutrias, body weight can affect the complexity of the procedure, particularly in surgeries requiring extensive internal manipulation, such as ovariectomy. In heavier animals, visceral fat accumulation may obstruct surgical access and make tissue visualization more difficult, leading to a prolonged surgery time (43). Additionally, greater body weight is often associated with an increased risk of anesthetic complications, which can further contribute to a longer overall surgery duration (44). In our study the interaction between weight and surgery approach was significant for surgery time. Specifically, as can be observed from Figure 5, for VMA surgery time and consequently anesthesia time increased while weight increased, whereas for FA the weight did not have any effect on surgery time. This result likely reflects the increased technical difficulty associated with surgery in heavier animals. This finding is consistent with previous studies suggesting that animals with greater body mass may require longer and more complex surgical procedures, not only because of tissue manipulation challenges but also to ensure adequate anesthetic management (42, 44). These results highlight the importance of considering body weight as a critical factor in planning and managing surgical procedures in rodents (41, 42, 44).

## Conclusions

Our observations indicate that using a lateral approach is faster and involves less manipulation of other viscera than the midline standard approach. This technique offers advantages such faster recovery, a key point in wild animal. Therefore, we propose it as a routine method for spaying clinically healthy—nutrias if managed in a confined sanctuary.

## Data Availability

The raw data supporting the conclusions of this article will be made available by the authors, without undue reservation.
