# Supplementary figures and images for: Midline vs. lateral flank approach for spaying nutrias (Myocastor coypus)
Source: Front Vet Sci. 2025 Jan 29;12:1529359. doi: 10.3389/fvets.2025.1529359 (PMC11813872; doi:10.3389/fvets.2025.1529359)

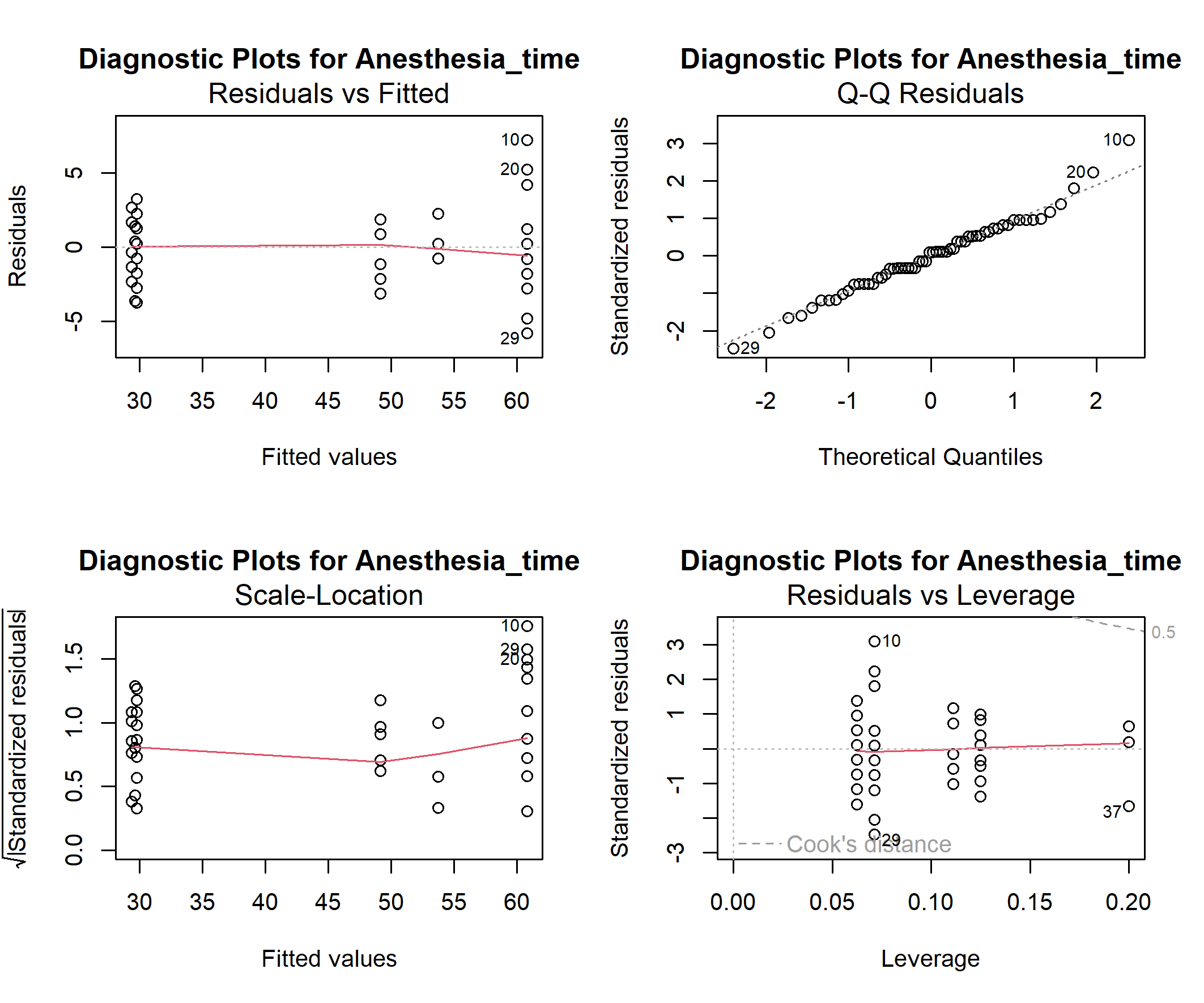

Supplement: Supplementary file 1 [file Image_1.png]

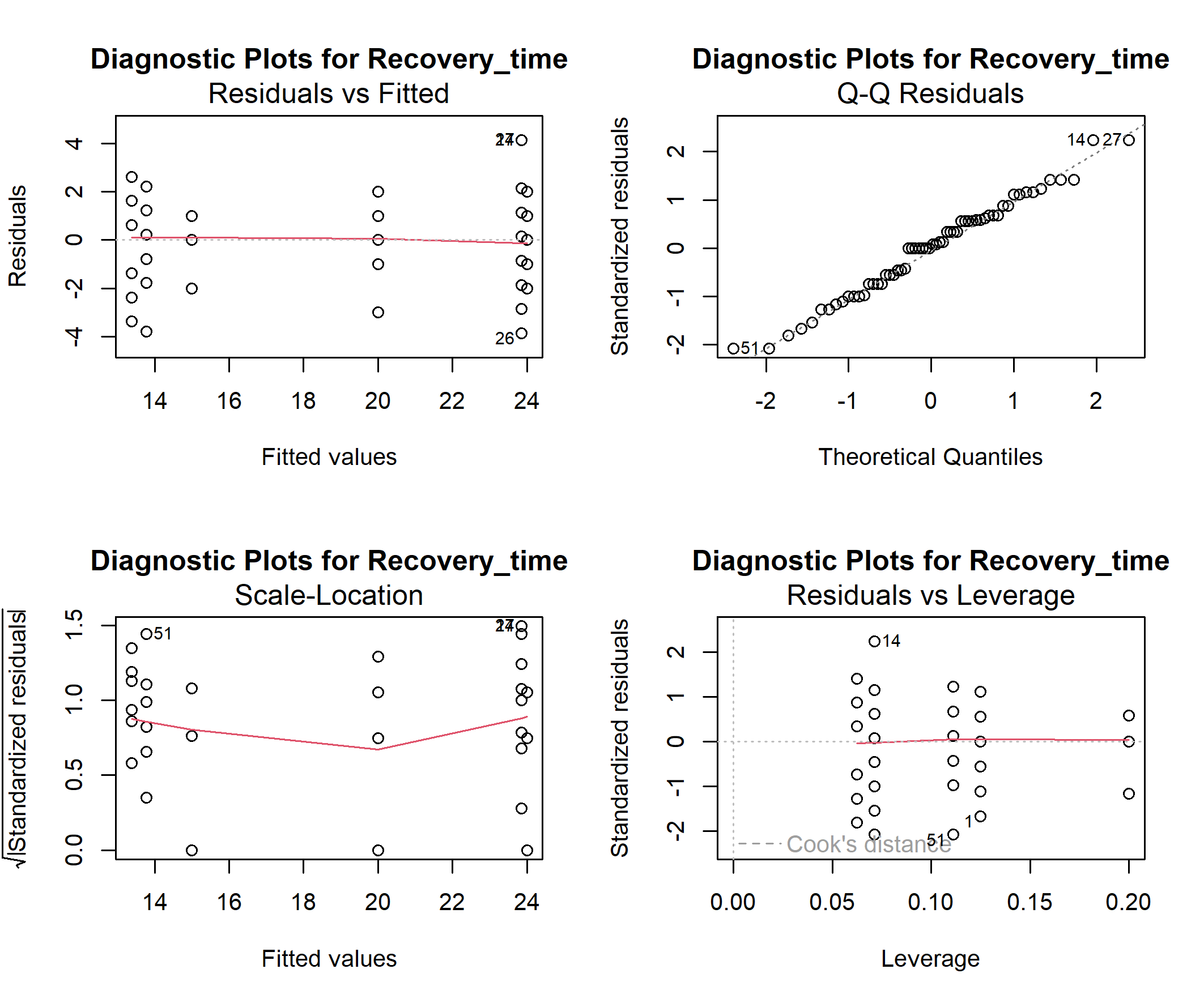

Supplement: Supplementary file 2 [file Image_2.png]

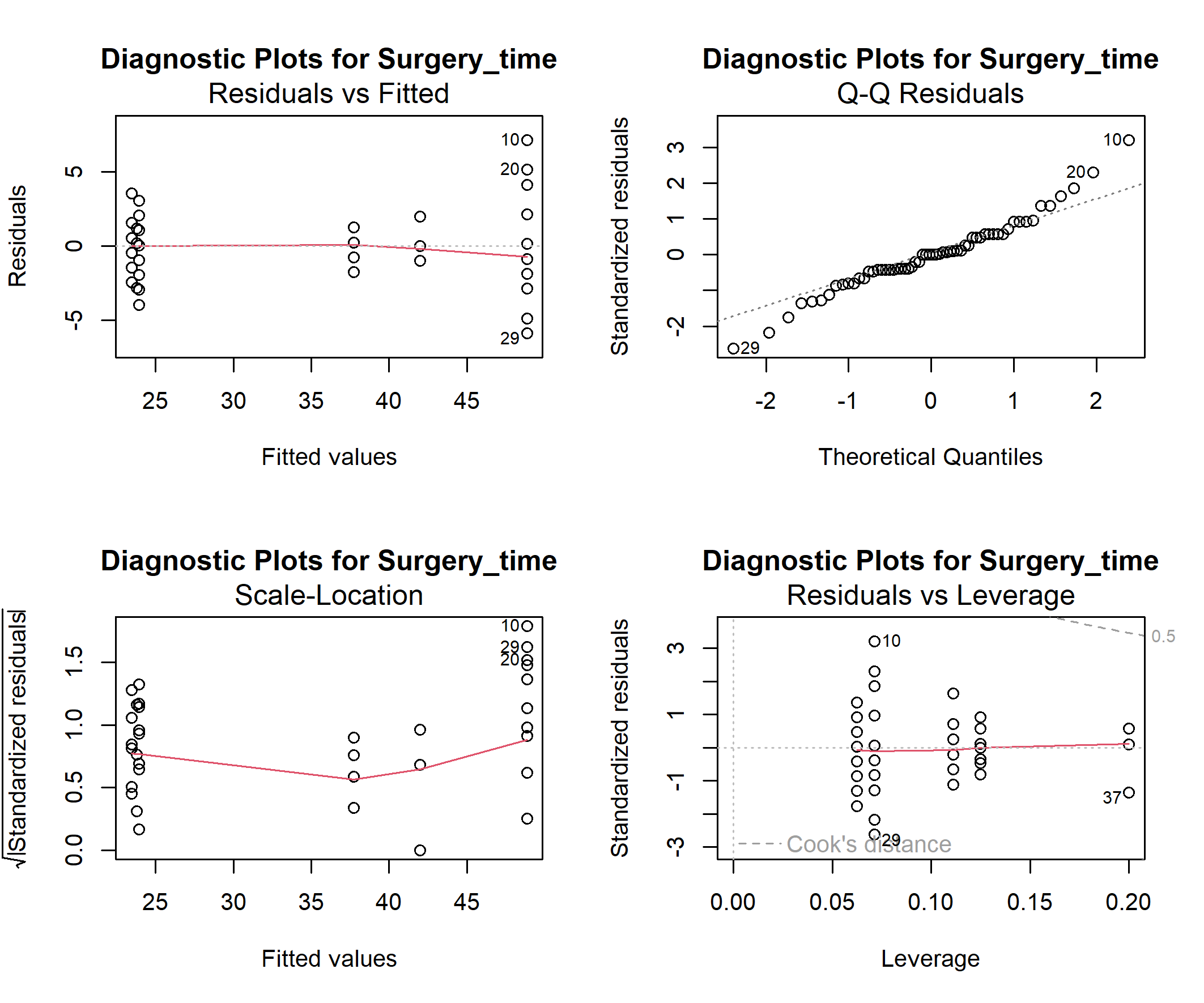

Supplement: Supplementary file 3 [file Image_3.png]

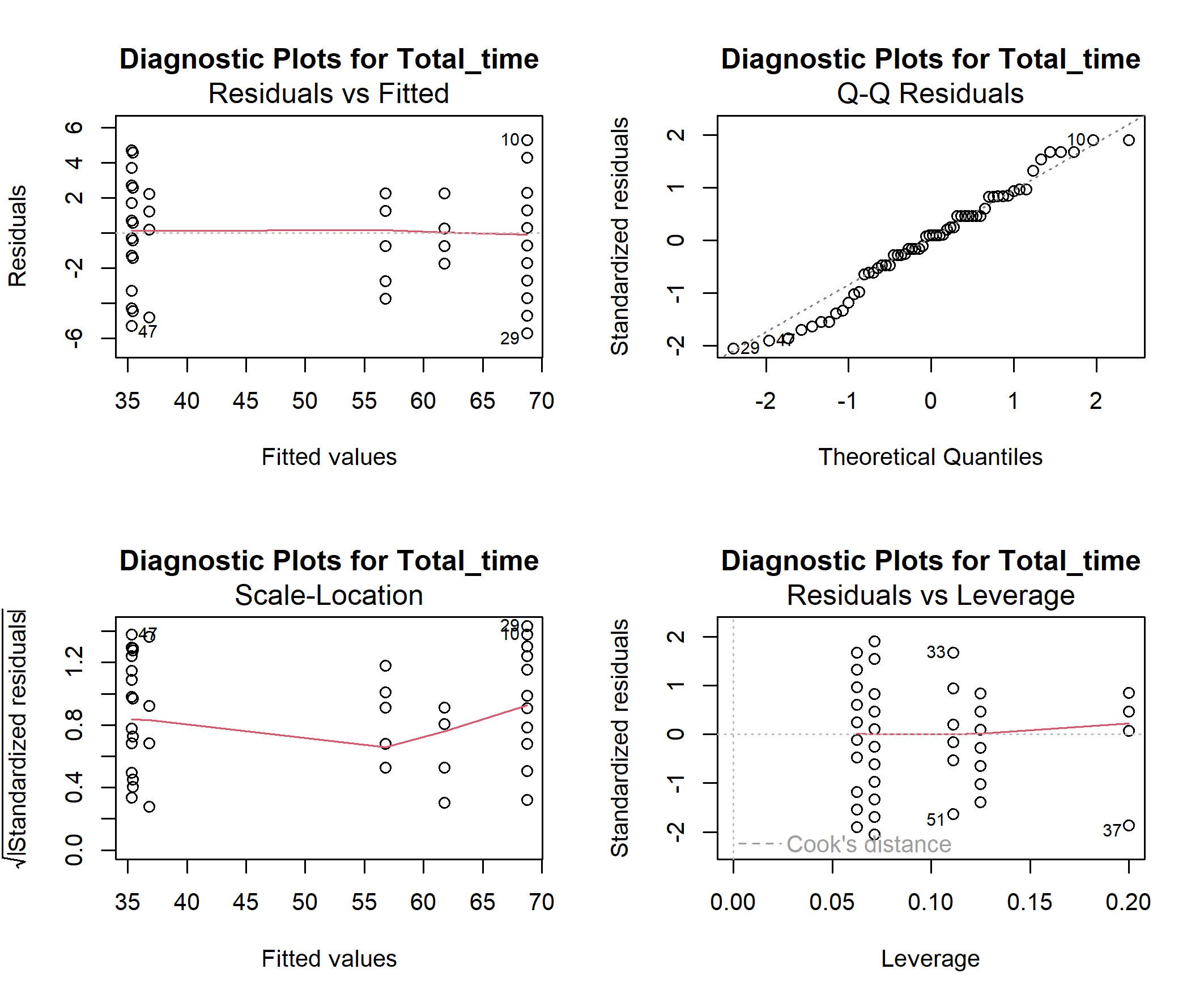

Supplement: Supplementary file 4 [file Image_4.png]
